# The PDE1/5 Inhibitor SCH-51866 Does Not Modify Disease Progression in the R6/2 Mouse Model of Huntington’s Disease

**DOI:** 10.1371/currents.hd.3304e87e460b4bb0dc519a29f4deccca

**Published:** 2014-02-13

**Authors:** Vahri Beaumont, Larry Park, Arash Rassoulpour, Ulrike Dijkman, Taneli Heikkinen, Kimmo Lehtimaki, Outi Kontkanen, Rand Al Nackkash, Gillian P. Bates, Melanie Gleyzes, Esther Steidl, Sylvie Ramboz, Carol Murphy, Maria G. Beconi, Celia Dominguez, Ignacio Munoz-Sanjuan

**Affiliations:** CHDI Management/CHDI Foundation, Los Angeles, California, USA; CHDI Management/CHDI Foundation, Los Angeles, California, USA; Brains On-Line LLC, South San Francisco, California, USA; Brains On-Line LLC, South San Francisco, California, USA; Charles River Discovery Research Services, Kuopio, Finland; Charles River Discovery Research Services, Kuopio, Finland; Charles River Discovery Research Services, Kuopio, Finland; Department of Medical and Molecular Genetics, Kings College London, London, UK; Department of Medical and Molecular Genetics, Kings College London, London, UK; Neuroservice, Domaine de Saint Hilaire, 13593 Aix en Provence cedex 03, France; Neuroservice, Domaine de Saint Hilaire, 13593 Aix en Provence cedex 03, France; PsychoGenics Inc., Tarrytown, New York, USA; PsychoGenics Inc., Tarrytown, New York, USA; CHDI Management/CHDI Foundation, Los Angeles, California, USA; CHDI Management/CHDI Foundation, Los Angeles, California, USA; CHDI Management/CHDI Foundation, Los Angeles, California, USA

## Abstract

Huntington’s disease is a neurodegenerative disorder caused by mutations in the CAG tract of huntingtin. Several studies in HD cellular and rodent systems have identified disturbances in cyclic nucleotide signaling, which might be relevant to pathogenesis and therapeutic intervention. To investigate whether selective phosphodiesterase (PDE) inhibitors can improve some aspects of disease pathogenesis in HD models, we have systematically evaluated the effects of a variety of cAMP and cGMP selective PDE inhibitors in various HD models. Here we present the lack of effect in a variety of endpoints of the PDE subtype selective inhibitor SCH-51866, a PDE1/5 inhibitor, in the R6/2 mouse model of HD, after chronic oral dosing.

## Introduction

Phosphodiesterases (PDEs) catalyze the degradation of cyclic nucleotides, and their inhibition leads to sustained signaling mediated by increased levels of cAMP, cGMP, or both. Intracellular cyclic nucleotide signaling plays a fundamental role in synaptic transmission, plasticity, and gene regulation 1 ^-^ 3. Specifically, signaling pathways downstream of cAMP elevation have been shown to be deregulated in Huntington’s disease (HD) models and in post-mortem samples obtained from HD patients 4 ^-^ 14 . Pharmacological treatment of HD mouse models with rolipram (a PDE4 selective inhibitor) and TP-10 (a selective PDE10 inhibitor) have been reported to significantly delay disease progression 5 ^,^ 7 ^,^ 14 ^,^ 15. PDE4 is a cAMP-specific PDE, whereas PDE10 modulates signaling by both cAMP and cGMP 3 ^,^ 16 ^-^ 18. Interestingly, PDE10 is one of the earliest and most significantly differentially down-regulated transcripts in many HD animal models and this downregulation is also apparent in post-mortem samples from HD patients 7 ^,^ 19 ^-^ 22. Whether this down-regulation of transcript expression constitutes a functionally meaningful event is unknown. It is possible that loss of PDE10 expression is a compensatory mechanism downstream of synaptic alterations in basal ganglia circuitry as PDE10 is strongly expressed in medium spiny neurons (MSNs) of the striatum 20 ^-^ 22. PDE1 is a calmodulin-dependent PDE encoded by 3 distinct genes (PDE1A, 1B and 1C) which include multiple splice isoforms. PDE1 hydrolyzes both cAMP and cGMP, and its activated state is regulated by cAMP-dependent protein kinase 23 ^-^ 25. It is widely expressed in brain, heart, and other tissues. However, the function of PDE1 in the brain has not been extensively investigated. Unlike PDE10, its levels seem to be only modestly downregulated in HD models and postmortem brain 26.

Based on studies showing that various PDE inhibitors have some benefit in the R6/2 model and that cAMP elevations may be associated with beneficial effects in neurons, we have begun a systematic evaluation of the efficacy of PDE inhibitors to uncover any potential for disease modification in HD models. We have synthesized or procured from industrial collaborators a set of brain-penetrant, selective PDE inhibitors for use as proof-of-concept molecules for the treatment of HD. Our criteria for selection are: molecules need to be selective for a PDE enzyme (to query the target), devoid of significant off-target activities in other protein targets of interest for brain function, and must display a suitable pharmacokinetic (PK) profile, including sufficient brain penetration, for chronic administration in mice. In this report we detail the evaluation of a relatively selective PDE1 inhibitor, SCH-51866 27 ^,^ 28, for its ability to modulate cAMP and cGMP levels in the striatum of R6/2 mice, and for its effects after chronic oral administration in a variety of endpoints of relevance to HD pathophysiology, including motor, cognitive, and structural endpoints. We show that despite a favorable pharmacokinetic profile to centrally inhibit PDE1 and strong evidence that this is achieved at oral doses >10 mg/kg, chronic administration of SCH-51866 did not ameliorate disease progression in the R6/2 model 29.

## Materials and Methods

**Analysis of SCH-51866 activity against PDE enzymes**

Studies were conducted at Scottish Biomedical (Scotland, UK)

Activity against recombinant PDEs (Table 1) was evaluated using the IMAP technology, which is based on high affinity binding of phosphate by immobilized metal coordination complexes on nanoparticles. The binding reagent complexes with phosphate groups on nucleotide monophosphate generated from cyclic nucleotides (cAMP/cGMP) through PDE activity. With fluorescence polarization detection, binding causes a change in the rate of the molecular motion of the phosphate bearing molecule, and results in an increase in the fluorescence polarization value observed for the fluorescent label attached to the substrate.

**Table 1 - Selectivity Profile of SCH-51866 against a PDE 1-11 panel.** NI, no inhibition noted at concentrations tested (up to 10 µM at Cerep and up to 100 µM at Scottish Biomedical) * Extrapolated value

Enzyme	SCH-51866 IC_50_(µM)
PDE1A3 (cAMP)	0.07
PDE2A3	1.32
PDE3 Cat	17.27*
PDE4 Cat	>100
PDE5 Cat	2.84
PDE6AB	NI
PDE7A1	>100
PDE8A1	NI
PDE9A2	51.35*
PDE10A1 (cAMP)	16.03*
PDE11A1 (cAMP)	21.89*

**Selectivity assays against additional CNS-relevant targets**

Studies were conducted at Cerep (France)

SCH-51866 was screened at 10 µM against a panel of PDEs (1-11; Table 1) and an additional set of >70 targets of relevance to the CNS (Cerep Diversity Profile); this latter analysis goes beyond the scope of the study presented here (data not shown). Assays were either binding or cellular assays, and conducted in duplicate wells.

**Electrophysiological evaluation in R6/2 hippocampal slices**

Electrophysiology was conducted at Neuroservice SARL, France. All animals were group housed under environmentaly enriched conditions and used in accordance with European legislations for animal care. On the day of recording, 7-8 week old R6/2 or wild type littermate (WT) mice were decapitated without anaesthesia followed by rapid brain removal into ice-cold oxygenated buffer (in mM; saccharose, 250, KCl 2, NaH_2_PO_4_ 1.2, MgCl_2_ 7, CaCl2 0.5, NaHCO_3_ 26, glucose 11). Hippocampal slices (350 μm thickness) were prepared with a MacIlwain tissue-chopper and incubated at room temperature (RT, 18 – 22 ^o^C) and allowed to recover for at least 1 h prior to recording in oxygenated Artificial Cerebro-Spinal Fluid (ACSF) of the following composition: (in mM; NaCl, 126, KCl 3.5, NaH_2_PO_4_ 1.2, MgCl_2_ 1.3, CaCl_2_ 2, NaHCO_3_ 25, D-glucose 11, pH 7.2). For experiments, individual slices were transferred to a 3-dimensional 60 electrode MEA chip (Ayanda Biosystems, Lausanne, Switzerland) and continuously perfused with oxygenated (95% O_2_–5% CO_2_) ACSF at 37^o^C at a rate of 3 mlmin^-1^, allowing for complete solution exchange within the MEA chamber 20 s following solution switches (MEA chamber volume: ~1 mL).

For stimulation, one MEA electrode underlying the Schaeffer collaterals at the CA3/CA1 interface was chosen visually as the stimulating electrode. The stimulus consisted of a monopolar biphasic current pulse (negative for 60 μs and then positive for 60 μs) applied at a frequency of 0.03 Hz. Recordings of resultant field excitatory postsynaptic potentials (fEPSPs) from electrodes underlying the CA1 region were made using MultiChannel Systems MCS GmbH software (MC Stim, MS Rack) and hardware composed of a 4-channel stimulus generator and a 60-channel amplifier head-stage connected to a 60-channel A/D card (Reutlingen, Germany). After stable baseline fEPSP amplitudes were achieved at 0.03 Hz stimulation frequency, an input-output (I/O) curve was performed on each slice to define basal evoked-responses for stimulation intensities between 100 and 800 µA, in 100 µA steps. Paired pulse facilitation (PPF) was then assessed at 40 % of the maximal fEPSP amplitude response (as defined from the I/O curve) by delivering two pulses to the stimulating electrode with decreasing inter-stimulus intervals (ISI of 300 ms, 200 ms, 100 ms, 50 ms, 25 ms). PPF was performed 4 times per slice, and the resultant 4 fEPSP amplitudes at a given ISI were averaged together. Slices were then exposed for 15 minutes to SCH-51866 (3 µM), before a second I/O and PPF protocol was applied in the continuous presence of compound. Thus I/O characteristics and PPF could be compared within the same hippocampal slice, before and after exposure of compound.

For measurement of long term potentiation (LTP), basal fEPSPs were recorded at 0.03 Hz for ten minutes at 40 % of maximal FEPSP amplitude to verify the baseline stability. LTP was then induced by a strong theta burst induction paradigm (TBS) consisting of a single train of 10 bursts composed each of 4 stimuli at 100 Hz, applied with a 200 ms interval. Potentiation of fEPSP amplitude was then monitored for an additional 60 minute period. In the case of treated slices, the whole LTP recording session was performed in the presence of 3 µM SCH51866 applied for 20 mins prior to TBS, and compared to slices perfused with ACSF containing only 0.1% DMSO vehicle perfused for a similar amount of time.

For each slice, the fEPSP values at each electrode recorded within the CA1 region were averaged to obtain a single fEPSP value per slice per timepoint. Data from experiments in slices carried out in the same conditions (genotype and treatment) were then averaged, and presented as mean ± SEM. Each treatment –genotype condition represents the mean of between 10 – 12 slices. For LTP analysis, the averaged fEPSP amplitude per slice was normalised to the pre-TBS amplitude. Data was analysed using Repeat Measure Two-way ANOVA followed by a post hoc Bonferroni test to analyse statistical significance of single data points using Prism 5.0 software.

SCH51866 was prepared as a 3 mM stock solution in DMSO, aliquoted and stored at -20°C. On the day of recording, a single aliquot of stock solution was diluted into ACSF to reach achieve 3 µM concentration, with a final concentration of 0.1% DMSO.

**RNA Extraction and Taqman**

Total RNA was extracted with the mini-RNA kit accordingly to manufacturer instructions (Qiagen). Reverse transcription (RT) was performed using MMLV superscript reverse transcriptase (Invitrogen) and random hexamers (Operon) and all Taqman-qPCR reactions were performed using the Chromo4 Real-Time PCR Detector (BioRad) as described. Expression level of the gene-of-interest was normalised to the geometric mean of three endogenous housekeeping genes (Primer Design) as described. Total RNA was extracted from brain tissue with the mini-RNA kit accordingly to manufacturer instructions (Qiagen). Reverse transcription (RT) was performed using MMLV superscript reverse transcriptase (Invitrogen) and random hexamers (Operon) and all Taqman-qPCR reactions were performed using the Chromo4 Real-Time PCR Detector (BioRad) as described 30. Expression level of the gene-of-interest was normalized to the geometric mean of three endogenous housekeeping genes (Primer Design) as described 30.

**Seprion ligand based ELISA method to monitor aggregate load**

Aggregates were captured in Seprion ligand coated plates (Microsens) and detected using the S830 sheep polyclonal or MW8 mouse monoclonal antibodies as described 9.

**Pharmacokinetic analysis in mice**

All PK studies were conducted at Charles River Laboratories (CRL), MA, USA. CRL is fully accredited by the Association for Assessment and Accreditation of Laboratory Animal Care International (AAALAC), and is registered with the United States Department of Agriculture (USDA). All procedures involving animals were conducted humanely and were performed by trained and experienced personnel. The individual study protocols were reviewed and approved by the IACUC of CRL.

Animals and Housing

A total of 48 male C57BL/6NCRL mice were used. Animals were acclimated to the study room environment for 5 days prior to dose administration. Mice were individually housed in micro isolator cages, and were kept on a 12/12 hour light/dark cycle except when interrupted for study procedures. Mice were fed LabDiet Certified Rodent Diet 5002 food ad libitum, except during fasting prior to dose administration, and had access to water ad libitum.

Mice were dosed with SCH-51866 at 5 mg eq./kg intravenously (iv) and at 10 mg eq./kg by oral gavage (po). For the iv doses, the compound was formulated at 1 mg/mL as a dose solution in 2% n-Methyl Pyrrolidine (NMP), 14% Propylene Glycol (PG), 14% Solutol HS–15, 70% 50mM Citrate, pH 4.5 , by volume. For the po dose, the compound was formulated at 1 mg/mL as a solution in 30% PG, 20% Solutol HS–15, 50% 50mM Citrate, pH 4.5, by volume.

Extraction and Bioanalysis

Aliquots (25 μL) of samples, plasma, and tissue blanks (containing internal standard only), plasma and tissue double blanks (without internal standard), control blanks (solvent only), diluted dose (dilute in plasma from the study species prior to extraction) and matrix calibration standards were dispensed into 96-well plates. Extracting solution with internal standard (100 μL) was added to all samples except matrix double blanks and solvent blanks. Extracting solution without internal standard (100 μL) was added to matrix double blanks. Samples were vortexed and centrifuged for 5 minutes, and the supernatants were transferred to a new plate. An aliquot (50 μL) of Milli-Q water was added to the samples, which were covered and vortexed for 5 minutes. Concentrations of SCH-51866 in mouse plasma and brain were determined using an LC-MS/MS assay. The lower limit of quantification (LLOQ) for this assay was 2.57 nM eq. for plasma and 0.642 nM eq. for brain (the limit of quantification was adjusted as applicable after applying the appropriate dilution factor). Calibration standards (1.00 – 10000 ng eq./mL for plasma and dose formulation; 0.250 – 2500 ng eq./mL for brain) were prepared by adding SCH-51866 to control mouse plasma or control mouse brain homogenate, respectively. Reserpine was used as an internal standard in the LC-MS/MS assay.

Mice were divided into four groups of 3 animals each. Two blood samples were collected from each mouse following dosing; the second collection was a terminal sample and brains were also collected at that time as follows: Group 1 at 0.083 and 0.5 hr, Group 2 at 0.25 and 2 hr, Group 3 at 1 and 8 hr and Group 4 at 4 and 24 hr., and brain tissue time points at 0.5, 2, 8 and 24 hr.

Non-compartmental pharmacokinetic analyses of the plasma concentration time course were performed using WinNonlin, version 5.2 (Pharsight Corp., Mountain View, California).

**Pharmacodynamic evaluation: effects in cGMP levels**

Microdialysis PK/PD studies were conducted at Brains Online, Inc. (San Francisco, CA, USA)

Microdialysis experiment

Male R6/2 (CBA/C57Bl/6 strain, CAG 120) mice aged 7-8 weeks (20 - 30 g; The Jackson Laboratory, USA) were used for the experiments. The animals were group housed in plastic cages (3-4 animals / cage) and had access to food and water *ad libitum*. Experiments were conducted in accordance with the declarations of Helsinki and were approved by the Institutional Animal Care and Use Committee of the University of California, San-Francisco.

SCH-51866 was formulated in a vehicle of 30% propylene glycol, 20% Solutol and 50% 50 mM citrate solution (pH 4.5). Mice were anesthetized using isoflurane (2%, 800 mL/min O_2_). Bupivacain/epinephrine was used for local anesthesia. Carprophen was used peri-/post-operative as analgesia. The animals were placed in a stereotaxic frame (Kopf instruments, USA), and microdialysis probes were inserted into the striatum. Coordinates for the tips of the probes were for striatum: posterior (AP) = +0.8 mm to bregma, lateral (L) = -1.7 mm to midline and ventral (V) = -4.0 mm to dura, the toothbar was set at 0.0 mm (Paxinos and Franklin, 2001). The microdialysis probes were either MetaQuant (MQ)probes (cellulose membrane, 3 mm exposed surface; BrainLink, the Netherlands) for the collection of dialysate used to measure the amount of SCH-51866 in the extracellular space following dosing, or I-shaped probes (polyacrylonitril membrane, 3 mm exposed surface; BrainLink, the Netherlands) for the analysis of cGMP in the dialysate following SCH-51866 dosing. After surgery animals were kept individually in cages and provided food and water *ad libitum*. Animals were fasted the night before the experiment.

Experiments were performed one day after surgery. On the day of the experiment, the probes of the animals were connected with flexible PEEK tubing to a microperfusion pump (Syringe pump UV 8301501, Univentor, Malta). MQ probes were perfused with aCSF containing 0.2% albumin at a flow rate of 0.10 µL/min. As the carrier flow, ultrapurified H_2_O was used at a flow rate of 0.70 µL/min. I-shaped probes were perfused with aCSF at a flow rate of 1.5 µL/min. Microdialysis samples were collected for 30-minute periods by an automated fraction collector (CMA 142, Sweden) into mini-vials. Samples originating from I-shaped probes were collected into mini-vials already containing 15 µL of 0.02 M formic acid. 10 µL of 0.02 M formic acid was added in the samples originating from MQ probes. Four basal samples (for pharmacodynamic study with conventional probes) or 3 basal samples (for pharmacokinetic study with MQ probes) were collected before the administration of SCH-51866. Following compound administration, microdialysate samples continued to be collected for 240 min at 30 minute intervals. All dialysis samples were stored at -80 °C.

After the experiment, the mice were sacrificed and plasma, brain tissue and tail tip samples were collected from each animal. For the plasma samples, blood was collected from the animal by heart puncture under isoflurane anesthesia. The blood was collected in a 1.3 mL micro tube (ref. 41.1393.105, Sarstedt, Germany) already containing an additive carrier with Li-Heparin. The blood was centrifuged at 4000 rpm at 4 °C for 10 minutes. Supernatants were stored as plasma samples at -80 °C.

Quantification of cGMP 

Concentrations of cGMP were determined by HPLC with tandem mass spectrometry (MS/MS) detection. Standard samples were prepared in artificial dialysate matrix, consisting of 3 parts (v/v) aCSF mixed with 1 part 0.02M formic acid in ultrapurified H_2_O. Final concentrations of standard samples were 0.02, 0.05, 0.1, 0.2, 0.5, 1, 2, 5 and 10 nM. Quality control (QC) samples were prepared independently. Final concentrations were 0.3 (QClow), 0.75 (QCmid) and 7.5 (QChigh) nM.

20 µL of each sample was injected into the LC system by an automated sample injector (SIL-20AD, Shimadzu, Japan). Chromatographic separation was performed on a Thermo BDS Hypersil C18, 2.1 * 150 mm column, held at a temperature of 30 °C. Components were separated using a linear gradient of ACN/0.1% FA in ultrapurified H_2_O/0.1% FA (flow rate 0.2 mL/min). The flow of the LC was diverted to the waste for 4.2 minutes, after which it was switched to the MS for detection of the analyte. MS analysis was performed using a API 4000 MS/MS system consisting of a API 4000 MS/MS detector and a Turbo Ion Spray interface (both from Applied Biosystems, USA). The acquisitions were performed in positive ionization mode, with ion spray voltage set at 5.5 kV with a probe temperature of 600 °C. The instrument was operated in multiple-reaction-monitoring (MRM) mode. For calibration of SCH-51866, the peak area ratio of the analyte versus universal internal standard was set against the analyte concentration. For calibration of cGMP, the peak area of the analyte was set against the analyte concentration. Curve fitting was performed by weighted (1/x) linear regression in the Analyst 1.4.2 software.

Statistical evaluation was performed in SigmaPlot for Windows, Version 11.0 (Systat Software, Inc., 2008).

The evaluation of SCH-51866 concentration in the microdialysis samples were performed based on Area under the curve (AUC) values. AUC were determined by the linear trapezoidal method between t = 0 and t = 240 with baseline subtraction. Treatment effects were evaluated among different drug concentrations and also compared to baseline, using two-way ANOVA for repeated measurements followed by Student-Newman-Keuls post-hoc test. Dose and time were the main effects. The level of statistical significance was set at p < 0.05. Pharmacodynamic parameters were calculated using Phoenix™ WinNonlin^®^ Pro Node initial (Pharsight Corporation, Mountain View, CA, USA).

**Behavioral, survival and MRI testing in R6/2 mice**

All *in vivo* behavioral, survival and MRI studies in R6/2 up to 30 mg/kg SCH-51866 reported in this manuscript were conducted at Charles River Discovery Research Services (Kuopio, Finland). An equally powered, separate full behavioral efficacy study at doses up to 3 mg/kg SCH-51866 was previously performed at Psychogenic, Inc. (Tarrytown, NY, USA) (data available in Supplementary Report 1). There was no improvement noted in any endpoint in this earlier study (rotarod, grip strength and open field evaluation, as well as survival).

Animals

All animal experiments were carried out according to the National Institute of Health (NIH) guidelines for the care and use of laboratory animals, and approved by the National Laboratory Animal Board, Finland. 60 male and female R6/2 TG mice (R6/2J CAG 120 and 12 male and female wild-type littermate control mice were bred at National Animal Facility Center, Kuopio, Finland.

Husbandry

All the mice were housed in groups of up to 4 per cage (mixed genotypes, single sex), in a temperature (22±1°C) and humidity (30-70%) controlled environment with a normal light-dark cycle (7:00-20:00). All mice were housed in shoe-box cages with clean bedding covering the ground that is changed as frequently as needed to provide the animals with dry bedding. This basic environment was enriched with the addition of play tunnels, wooden nesting material, and plastic bones for all mice; i.e. an environmentally-enriched cage contains a Mouse Tunnel, (amber color, certified, transparent, BioServ Product# K3323) and a Petite Green Gumabone (BioServ Product # K3214) and wooden nesting material. Food (Purina Lab Diet 5001) and water were available ad libitum to the mice in their home cages. R6/2 and corresponding WT mice also receive wet powdered food (Purina Lab Diet 5001 mixed with water to form a paste) placed inside a cup on the floor of the cage; this additional food was replaced fresh daily and started from the time of weaning. In addition, water spouts were fitted with extensions to allow mice to easily access from floor level.

Experimental Set Up of Mice

In setting up groups for study (i.e. vehicle or compound treated), transgenic and wild-type mice were randomized into groups so that whole litters of mice did not end up in a single testing group. Mice were housed in groups of 4 and separated by sexes. In each cage, one wild-type mouse of the same sex, but different litter, was included in an attempt to provide normal social stimulation. Mice were allowed to acclimate to the experimental room for at least one hour prior to the beginning of any experiment. Mice were transported from the colony room to experimental rooms in their home cages. Experimentation was conducted in a blinded manner. Tail samples were taken at the end of the study for verification of genotypes and CAG sizes of individual mice.

Compound Delivery

**Table:** 

Genotype	N (F/M)	Compound	Dose (mg/kg)	Regimen	Route	Dose volume (ml/kg)
Wild-type	6/6	30% Propylene Glycol, 20% Solutol HS15, 50% 50mM Citrate		BID	p.o.	10
R6/2	10/10	30% Propylene Glycol, 20% Solutol HS15, 50% 50mM Citrate		BID	p.o.	10
R6/2	10/10	SCH-51866	10	BID	p.o.	10
R6/2	10/10	SCH-51866	30	BID	p.o.	10

Body Weight and Survival

Body weight was measured starting at the age of 4 weeks and a further two times per week on the same day until the end of the study. This was done just prior to animals receiving doses for those days. In the case that the general health status of an animal had significantly worsened, the mouse was sacrificed by CO_2_ asphyxiation. A mouse was euthanized if observed not to be able to eat or drink, if mouse did not respond to gentle stimulation or if mouse was unable to right itself in 30 sec period.

Neurological Index

The mice were observed for 1-2 minutes per mouse at 13 weeks of age. The following 30 behaviors were evaluated: Head Tremor, Head Twitch, Head Bobbing, Head Searching, Body Tremor, Body Twitch, Tail Tremor, Tail Twitch, Straub Tail, Piloerection, Shallow Respiration, Flattened Body Posture, Swollen Face, Ptosis, Irritability, Seizure, Urine Staining, Lacrimation, Salivation, Limb Splay, Catalepsy, Abnormal Gait, Tip Toe Walking, Slow Careful Movements, Excessive Grooming, Circling, Sniffing, and Chewing. In addition to the above occurrences the chronic observational phase includes excessive locomotor activity, loss of startle response, loss of righting reflex, dehydration and tail pinch. The assessment was performed as follows: A score of 0 was assigned for normal features (such as locomotor activity) or for the absence of abnormal features (such as absence of piloerection); a score of 1 was given when mild abnormalities were observed; and a score of 2-3 was given when severe abnormalities were observed. All of these features were scored from simple observation of the mice in their cage, except for the startle, tail pinch and righting reflexes which are direct manipulations. To test the startle reflex, a small hand clicker was used to generate a loud popping noise and the following behaviors were identified during this process: jumping, freezing, and rapid eye blinks. For the righting reflex, each mouse was then removed from its home cage and placed on its back allowing the mouse to correct itself. Tail pinch was tested by gently squeezing the end of the tail with forceps.

Motor Function

Motor function testing including open field and rear climbing tests were commenced at 4 weeks of age and continued at weeks 6, 8 and 12, for grip strength test at age weeks 4 and 12 and for rotarod at age weeks 4, 6 and 8. Compound treatment was started at 4 weeks of age after the baseline behavioral tests.

Rotarod

Mice were tested at age weeks 4, 6 and 8 over 3 consecutive days (Wed, Thu, Fri). Each daily session included a training trial of 5 min at 4 RPM on the rotarod apparatus (AccuScan Instruments, Columbus, USA). One hour later, the animals were tested for 3 consecutive accelerating trials of 6 min with the speed changing from 0 to 40 RPM over 360 seconds and an inter-trial interval at least 30 min. The latency to fall from the rod was recorded. Mice remaining on the rod for more than 360 s were removed and their time scored as 360 sec.

Grip Strength

Grip strength measurement was performed at age weeks 4 and 12. The mice were tested on Monday afternoons. Mice were taken to the experimental room and, one at a time, were placed on the grip-strength apparatus (San Diego Instruments, San Diego, USA) in such a way that the animal grabs a small mesh grip with its forepaws. The entire apparatus is placed on a table top for testing. Animals are lowered to the platform and then slowly pulled away from the handle by the tail until the animal releases the handle. The equipment automatically measures the strength of the animal’s grip in grams. Five scores were recorded per animal in consecutive sequence. Mice were returned to their home cage after testing.

Open Field Test

Open field test was performed at age weeks 4, 6, 8 and 12. The mice were tested on Mondays at approximately 10-30 lux of red light. The mice were brought to the experimental room for at least 1 h acclimation to the experimental room conditions prior to testing. Activity chambers (Med Associates Inc., St Albans, VT; 27 x 27 x 20.3 cm) are equipped with IR beams. Mice were placed in the center of the chamber and their behavior was recorded for 30 min in 5-minute bins. Quantitative analysis was performed on the following five dependent measures: total locomotion, locomotion in the center of the open field, rearing rate in the center, total rearing frequency and velocity.

Rear Climbing Test

Rear climbing test was performed at age weeks 4, 6, 8 and 12. The mice were tested on Tuesdays. Each animal was placed into custom made pencil holder, and was scored for 5 minutes. During this period the following events were noted: 1) number of times the mouse rears without touching the mesh walls, 2) latency to climb (lifting 4 paws from the floor is labeled climbing), 3) climbing time, and 4) climbing instances.

*Cognitive Function* *: Cued Two-Choice Swim Test*

The cued two-choice swim test was performed at 9 weeks of age. Testing was conducted under low light, white light conditions. The mice were taught to swim to (acquisition), or away from (reversal), a light positioned over the escape platform. Animals were placed within a rectangular tank (76 cm x 30 cm x 30 cm) filled with water maintained at 25°C ± 1°C. The water was rendered opaque by the addition of non-toxic white paint. The escape platform was located 0.5 cm below the surface of the water at one end of the tank. A light source with a 25W bulb (white light), oriented toward the water, was clipped to one side of the tank above the escape platform (7 cm in diameter with the borders slanted and roughened to help the rodents climb to it). Under these conditions, the light intensity is measured as 200 lux on the illuminated side of the tank, and 3 lux on the dark side of the tank. The platform was located on the lit side of the arena across acquisition trials, although the location of the lit side within the room relative to the mouse’s entry point was varied. The location of the bulb and platform was different on different trials for the same mouse. On each trial, mice were placed into the middle of the tank and allowed to swim for up to 60 sec, or until they found the platform. If an animal did not reach the platform within 60 sec, the mouse was placed on it by the experimenter and allowed to remain there for 30 sec and then removed to a pre-warmed holding cage placed on a warming pad. During all acquisition days, mice were given 8 trials per day (4 blocks of 2 trials, separated by an interblock interval of 20-30 minutes). Training continued in this way until each mouse performed at 75% correct choices for 2 consecutive days; upon reaching this criterion for successful acquisition of the task, mice were placed into the reversal phase on an individual basis. During reversal training, (4 blocks of 2 trials per day), the platform was placed on the dark side of the tank. On each trial during testing days, the latency to reach the platform was recorded (60 seconds if the platform was not reached) and the trial was scored as either “correct”, “incorrect” or “no choice”. The maximum number of days of acquisition was 12 days. All mice went from acquisition into the reversal phase and the experiment was stopped when 2 consecutive days of 75% criterion was reached or after a maximum of 7 days of reversal testing. A correct choice was scored if the animal turned in the direction of the platform and successfully mounted the platform. An incorrect choice was scored if the animal swam in the direction opposite the platform. No choice was scored if the animal either did not make a choice, by swimming in the middle of the tank for the 60 sec, or turned initially toward the platform but did not mount the platform. To make a choice, a mouse had to swim within 20 cm of either end of the tank.

MRI

MRI analysis was performed for all mice at the age of 13 weeks in a horizontal 7T magnet with bore size 160 mm (Magnex Scientific Ltd., Oxford, UK) equipped with Magnex gradient set (max. gradient strength 400 mT/m, bore 100 mm) interfaced to a Varian DirectDrive console (Varian, Inc., Palo Alto, CA) using a volume coil for transmission and surface phased array coil for receiving (Rapid Biomedical GmbH, Rimpar, Germany). Isoflurane -anesthetized mice were fixed to a head holder and positioned in the magnet bore in a standard orientation relative to gradient coils. Rectal temperature (TC-1000 Temperature Controller, CWE Inc., Ardmore, PA, USA) and respiration (ECG Trigger Unit, Rapid Biomedical GmbH) were monitored throughout the study and the body temperature was kept at 36-37°C. For determination of volume of brain, striatum, cortex and lateral ventricles T2-weighted multi-slice (17 continuous slices) images were acquired using fast spin-echo sequence with TR/TE_eff_ = 3000/36 ms, matrix size of 256x128, FOV of 20x20 mm^2^, slice thickness of 0.7 mm and 8 averages.

Statistical analysis

All values are presented as mean ± Standard Error of Mean (SEM), and differences are considered to be statistically significant at the p < 0.05 level. Statistical analysis was performed using StatsDirect statistical software. Differences between group means were analyzed by 1-way-ANOVA followed by Dunnett’s test (comparison to the control (=vehicle treated TG mice) group). The acquisition of the Cued two-choice swim test was analyzed using Fisher’s exact test.

## Results and Discussion

SCH-51866 ((+)-cis-5,6a,7,8,9,9a-hexahydro-2-[4-[trifluro-methyl] phenylmethyl]-5-methyl-cylopent[4,5]imidazo [2,1-b]purin-4(3H)one; Figure 1) is reported as a selective PDE1/5 inhibitor, developed initially for hypertension 27 ^,^ 28. In order to confirm its selectivity profile, we evaluated its potency against a panel of recombinant PDEs (Table 1). In brief, SCH-51866 was confirmed as a potent PDE1 inhibitor (IC50 70 nM), and in our assays displayed much weaker potency for PDE2A3 (IC50 1.32 µM) or PDE5 catalytic domain (IC50 2.84 µM). In broad agreement with these findings, SCH-51866 showed a complete inhibition of PDE1B (100% inhibition), PDE2A (92% inhibition) and PDE5 (88% inhibition) when tested at 10 µM in the Cerep diversity panel, consistent with previously reported potencies reported for SCH-51866 against various PDEs; 44 µM for PDE9 and 2 µM for PDE2 (12 ^,^ 23 ^,^ 25 ^,^ 27 ^,^ 28; see Table 1). However, SCH-51866 has been reported to be equipotent against PDE1 and PDE5 (reported IC50 of 70 and 60 nM, respectively 12 ^,^ 23 ^,^ 27 ^,^ 28). The reason for this discrepancy is unclear, although it may be dependent on using full length PDE5 versus the catalytic domain used in our assays (12 uses full length PDE5 derived from bovine lung). PDE5 is expressed in cerebellar Purkinje cells, and low-level expression is detected by northern blot in various nuclei of relevance to the pathology observed in HD, most prominently in subthalamic nucleus and substantia nigra 31 ^-^ 33. Based on the expression of PDE1 and 5 in brain regions of interest, the PDE selectivity profile for SCH-51866 was deemed acceptable to selectively query the therapeutic potential of PDE1 and 5 inhibition in HD mouse models. The Cerep diversity panel screen showed low off-target activity against other CNS-relevant targets, showing submaximal inhibition of only a further 4 targets when assessed at 10 µM; 88% inhibition at the α2-adrenergic receptor, 57% inhibition at NK3 receptor, 66% at the site 2 of the Na2+-channel, 94% inhibition at the human choline transporter. Based on our data, given the expected full inhibition of PDE1 inhibition at concentrations less than 1 µM, none of these off target effects would be expected to contribute to observed efficacy, as long as brain exposure of SCH-51866 was kept in the low µM range. Based on these results and published data, SCH-51866 appears as a relatively selective PDE1/5 inhibitor for us to evaluate the role of these target(s), and especially PDE1, in an HD context.

**Flow scheme of candidate proof-of-concept molecule evaluation as a potential therapeutic for HD in rodents.**
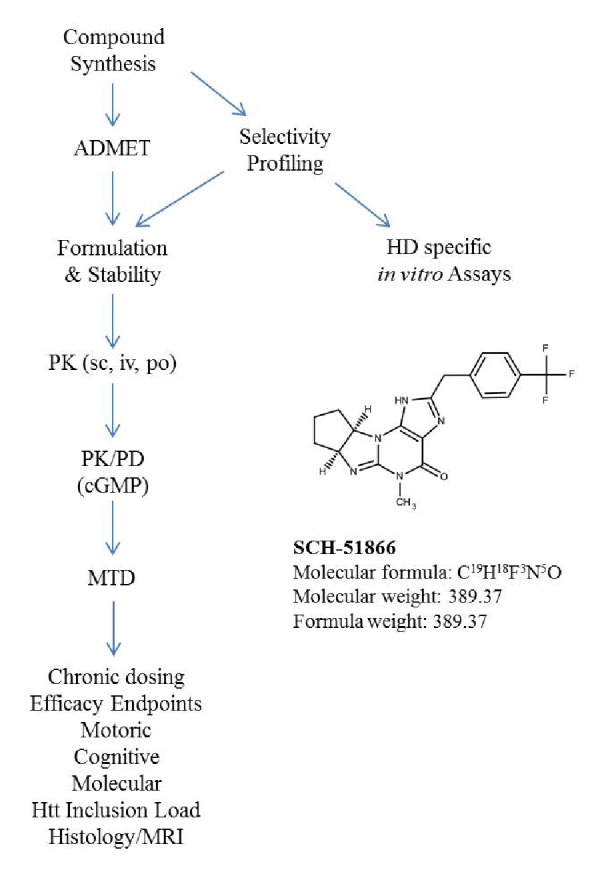
This flow scheme is representative of the CHDI compound testing process.

Electrophysiology

We have previously shown that R6/2 mice show alterations in hippocampal synaptic transmission, as well as short and long term plasticity, when evaluated ex vivo in acutely prepared brain slices (Beaumont et al; SFN 2009 abstract #433.22/K23). Elevations in basal synaptic transmission in the CA3:CA1 synapses are evident by 6 weeks of age, becoming increasingly more pronounced as the mice become overtly symptomatic. This elevated basal transmission (Figure 2a) is accompanied by a decrease in paired pulse facilitation (PPF) ratios (Figure 2b), suggesting that the elevated basal synaptic transmission is presynaptic in origin, most likely due to elevated glutamate release 34. Despite elevated basal synaptic transmission, long term plasticity, as assessed by long term potentiation (LTP), is impaired in R6/2 compared to WT (Figure 2c). These phenotypes have been seen in other HD models as well; elevated synaptic release has been observed in *Drosophila* models of HD and R6/1 mice at the neuromuscular junction 35, and impaired hippocampal LTP has been documented previously in R6/2 26 ^,^ 36, as well as in knock-in mouse models of HD 37 ^-^ 40.

**Evaluation of SCH-51866 in acute hippocampal slices.**
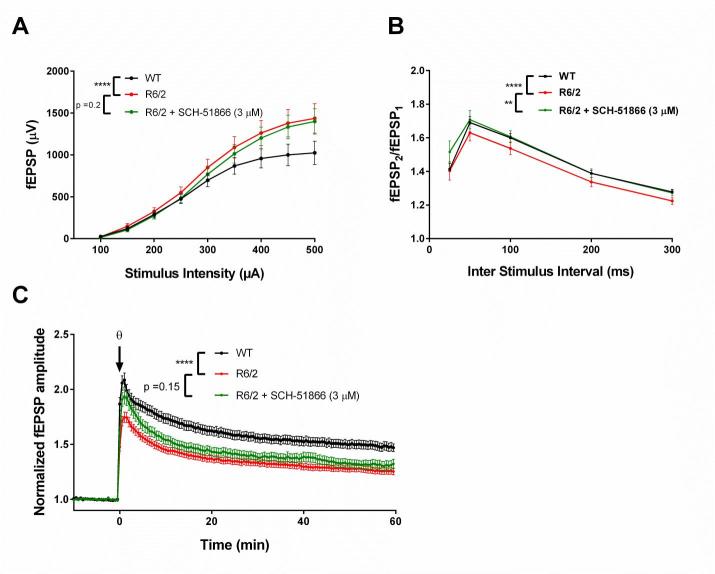
(A) Basal synaptic transmission is elevated in R6/2 compared to WT. SCH-51866 has no significant effect on R6/2 input/ouput (I/O) parameters; (B) SCH-51866 restored R6/2 paired-pulse facilitation (PPF) to WT levels; (C) SCH-51866 did not restore the deficit in R6/2 long-term potentiation (LTP). Significance is denoted by asterisks (p< 0.0001 = ****, p < 0.01=**)

To assess whether SCH-51866 was capable of acutely reversing any of these electrophysiological phenotypes in R6/2 hippocampal slices, we measured basal synaptic transmission at various stimulating intensities, followed by an investigation of PPF; firstly in the absence of SCH-51866, and then in the presence of 3 µM compound. SCH-51866 did not significantly reduce the elevated basal synaptic transmission as assessed by the I-O characteristics of the slices, but PPF was significantly restored to WT levels (Figure 2b). This indicates a modest reduction in glutamate release probability after PDE1 inhibition.

We next sought to investigate whether SCH-51866 could potentiate the impaired LTP that is observed in R6/2 as compared to WT slices. Application of SCH-51866 (3 µM) to R6/2 slices did not result in an amplitude of LTP significantly different to R6/2 slices recorded in ACSF alone (Figure 2c).

We conclude that SCH-51866 does not improve electrophysiological parameters in hippocampal slices from R6/2 animals, at a concentration sufficient to fully inhibit the PDE1 enzyme.

**Formulation and Pharmacokinetic of SCH-51866 analysis in mice**

To our knowledge, the PK analysis of SCH-51866 in mice has not been reported, although a rat PK study was reported 27. The PK analysis of SCH-51866 in mice is shown in Figure 3 and Table 2. Following intravenous (iv) administration, plasma clearance was moderate (1.75 L/hr/kg), the volume of distribution at steady state was high (4.48 L/kg) and the elimination half -life was 3.87 hours. Following an oral (po) dose, the compound was rapidly and well absorbed with C_max_ occurring within 1 hour and bioavailability of 61% (Table 2). Concentrations in brain were measurable for all animals through 8 hours following dosing. Brain-to-plasma concentration ratios (0.1 - 0.4) suggested good distribution of the compound into the brain parenchyma. At Tmax, the concentration of SCH-51866 in mouse brain was approximately 500 nM after oral administration (Table 2). This concentration effectively exceeds the biochemical IC50 for PDE1 by ~7-fold, but is not in the expected range for any known off-target activity. The plasma Cmax exposure at 10 mg/kg po in mice is similar to that reported for rats (2400 nM in our study; 2370-3800 nM in rats 27). Based on the half-life of the molecule in plasma (Table 2), we selected BID (twice a day) dosing for all efficacy studies in HD models. Given its acceptable PK profile and selectivity, we decided to perform an exposure/pharmacodynamic (PD) analysis of the SCH-51866 in adult R6/2 mice, to confirm adequate CNS target engagement.

**Pharmacokinetic (PK) analysis of SCH-51866 after acute administration in mice.**
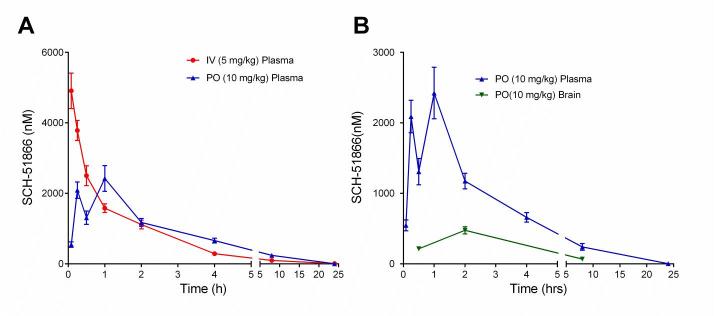
(A) Exposure of SCH-51866 in plasma following IV (5 mg/kg) or oral (10 mg/kg) administration; (B) Exposure of SCH-51866 in plasma and brain tissue after oral (PO) (10 mg/kg) dosing.

**Table 2 - PK analysis of SCH-51866 in mice following oral (po), subcutaneous (sc) or intravenous (iv) administration:** ^1^ Values were not calculated since there were not enough points in the elimination phase. NA: Not applicable PK parameters due to route and/or matrix. NC: Not calculated due to insufficient data points to define the corresponding PK parameter.

PK Parameters	Units	Plasma		Brain	
		IV 5.0 (mg eq./kg)	IV 5.0 (mg eq./kg)	IV 5.0 (mg eq./kg)	PO 10.0 (mg eq./kg)
AUC_(0-last)_	nM eq.·hr	7310	8960	8180	2200
AUC_(0-inf)_	nM eq.·hr	7350	8970	8230	NC
Bioavailability (F)	%	NA	61.0	NA	NA
Observed C_max_	nM eq.	NA	2420	3560	475
Observed T_max_	hr	NA	1	0.5	2
Plasma Clearance (CL_p_)	L/hr/kg	1.75	NA	NA	NA
Volume of Distribution at Steady State (Vd_ss_)	L/kg	4.48	NA	NA	NA
Mean Residence Time (MRT)	hr	2.57	NA	NA	NA
Half-life (t_1/2_)	hr	3.87	2.49	1.08	NC^1^
Half-life Regression Time Points	hr	4, 8, 24	4, 8, 24	0.5, 2, 8	NA

**Pharmacodynamic analysis of cGMP elevation following acute administration of SCH-51866**

****The use of a PD endpoint is a requisite step in our drug evaluation process. The interpretation of positive or negative results after chronic dosing necessitates a dynamic marker to understand dose proportionality of the intended biological response, as well as to evaluate the site of action of the molecule. Towards this goal, we evaluated SCH-51866 for its ability to increase cGMP in the striatum of wildtype (WT) and R6/2 animals. The selection of a rodent model is dependent on the mechanism being targeted, and in this case, based on expression profiling (unpublished observations), PDE1 appears to be expressed similarly in WT and R6/2 animals. This is in contrast to, for instance, PDE10 5 ^,^ 7 ^,^ 10, which is strongly downregulated as the disease progresses in HD models.

In brief, male R6/2 animals at 7-8 weeks of age were surgically implanted with a microdialysis probe in the striatum and administered a single oral dose of SCH-51866. Dialysate fluid obtained in 30 minute intervals were used to investigate the amount of compound present (Figure 4a), and the same doses were used in a separate cohort of animals to assess effects on cGMP accumulation in the extracellular matrix (Figure 4b). This allows a comparative PK/PD evaluation of SCH-51866 based on free drug exposure versus response in the brain region of interest. Figure 4 shows a dose-dependent accumulation of SCH-51866 in brain extracellular fluid, and a concomitant elevation of cGMP. The accumulation of cGMP increased dose-proportionally (Figure 4c), and highlighted that only at 10 and 30 mg/kg were we able to detect significantly elevated cGMP levels. Levels of SCH-51866 in dialysate are within the highly selective range for PDE1 inhibition (~ at or below biochemical IC50). Since PDE1 is an intracellular enzyme this gives an approximation of free levels achieved intracellularly which can inhibit target, assuming that the extracellular and intracellular concentrations are at steady state. Furthermore, elevation of cGMP in the extracellular matrix is proportional but not necessarily equal to the intracellular concentration of cyclic nucleotides, which are likely to be higher than those detected in the extracellular matrix. . Nevertheless, this experiment demonstrates that the 10 and 30 mg/kg po doses of SCH-51866 are sufficient to inhibit PDE1 centrally and to induce the expected elevation in cGMP. This was used as a benchmark to limit the top dose used in subsequent efficacy evaluation. However, as SCH-51866 has been reported to elicit peripheral effects in rats when dosed orally at lower concentrations (0.3-10 mg/kg 27), we also evaluated additional lower doses in our efficacy studies.

**Pharmacodynamic (cGMP) analysis after acute administration of SCH-51866 in male R6/2 mice.**
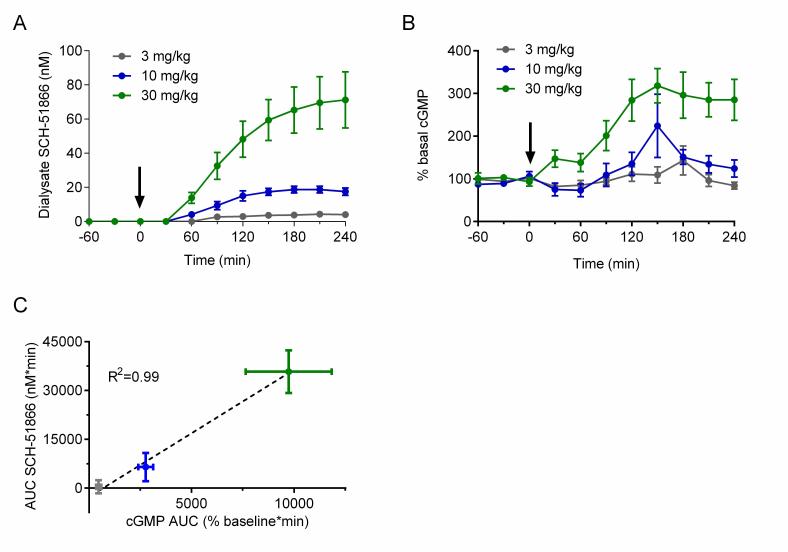
(A) Quantification of SCH-51866 concentration in extracellular dialysate after po acute administration; dialysate levels shown are not adjusted for recovery factor, which on average was 56%; (B) Quantification of cGMP elevation measured in extracellular dialysate after acute po administration; (C) Correlation of AUC evaluation of SCH-51866 and cGMP elevation in extracellular dialysate shows a dose-proportional increase in cGMP in response to increasing concentration of SCH-51866. Elevation in cGMP was significant over baseline for both 10 and 30 mg/kg dose groups, but not the 3 mg/kg dose group.

**Efficacy Evaluation of SCH-51866 in R6/2 mice**

Prior to chronic dosing, we conducted an acute and subchronic (2 weeks) tolerability evaluation in R6/2 mice starting at 6 weeks of age (studies conducted at Psychogenics, USA; see Supplementary Report 1 for full details). All doses administered were well tolerated with no sign of adverse effects, allowing us to proceed with the efficacy evaluation after chronic administration.

Based on the PK/PD analysis, we confirmed that 10 and 30 mg/kg SCH-51866 was sufficient to elevate cGMP centrally, confirming target engagement. We used these doses to investigate potential effects in a variety of endpoints in R6/2 mice. (We also investigated lower doses, 0.3 – 3 mg/kg, which had no effect in ameliorating disease progression in the R6/2 mice; see Supplementary Report 1 for all lower dose groups.)

A total of 60 male and female R6/2 mice (C57Bl/6, CBA background) and 12 male and female WT littermate control mice were used in the high dose study presented here. The BID p.o. treatment with SCH-51866 (10 or 30 mg/kg; Figures 5-8) or vehicle started at 4 weeks, immediately after baseline behavioral evaluation. Body weights were measured twice a week starting at week 4 and continued until death (Figure 5A). Grip strength tests (Figure 5B) were conducted at age weeks 4 and 12 and the rotarod test (Figure 5C) was conducted at age weeks 4, 6 and 8. Tests to assess motor function, including open field (Figure 6A-D) and rear climbing tests (Figure 6E, F), were conducted at 4, 6, 8 and 12 weeks of age. In addition, a cued two-choice swim test (Cued 2-CST) was performed at 9 weeks of age (Figure 7). At 13 weeks of age all the mice were subjected to in vivo T2-MRI analysis of brain, striatum and cortical volume (Figure 8A, B). The mice were followed until death (spontaneous death or fulfillment of euthanasia criteria). Survival data was recorded and graphed as Kaplan-Maier plot (Figure 8C). With the exception of a positive effect in improving grip strength when dosed at 30 mg/kg, SCH-51866 did not modify any R6/2 disease-related phenotypes (summary of all results in Tables 3 and 4; for further details see Supplementary Report 2).

**Behavioral evaluation during chronic administration of SCH-51866 in R6/2 mice.**
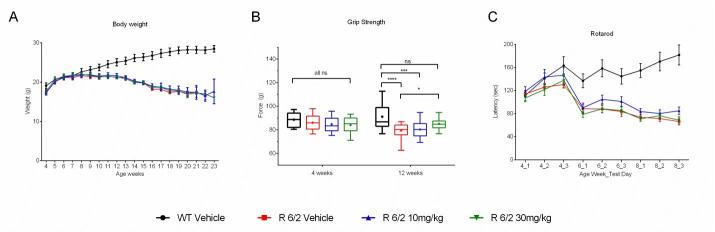
Analysis of (A) body weight; (B) Grip strength measured at 12 weeks of age was significantly improved over the R6/2 vehicle group in the 30 mg/kg dose group, and was equivalent to wild type vehicle controls; (C) Rotarod performance;

**Locomotor effects of chronic SCH-51866 in R6/2 mice.**
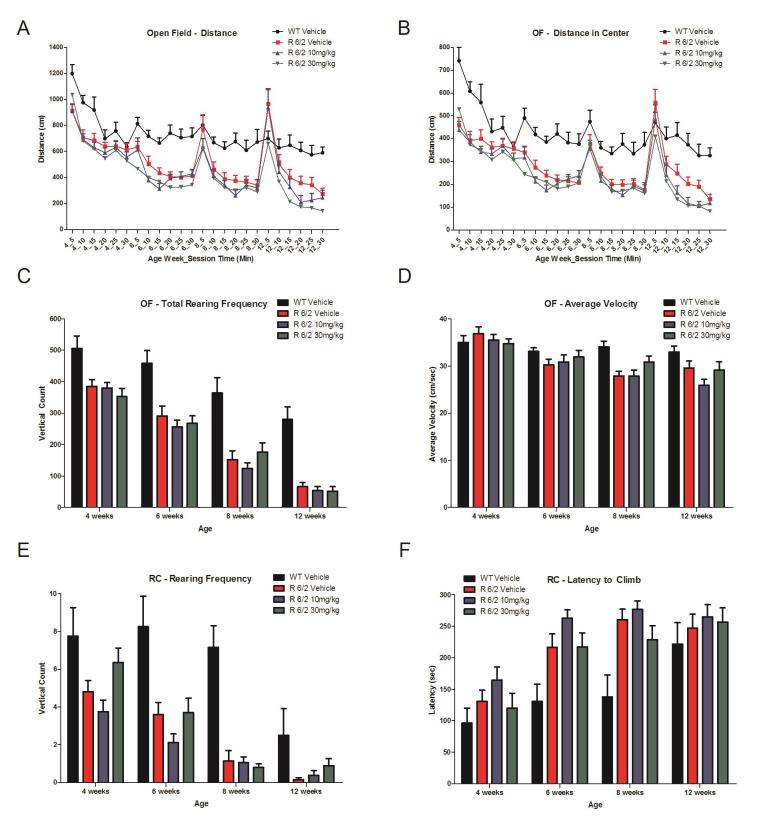
Chronic SCH-51866 administration in R6/2 mice showed no improvement in (A) Open field total distance traveled, (B) distance in center (C), total rear frequency, (D) average velocity in an open field, (E) in rearing frequency or (F) latency to climb a custom-made pencil holder.

**Lack of effect of SCH-51866 in a cognitive task (Cued Two-Choice Swim Test; C2CST) in R6/2 mice.**
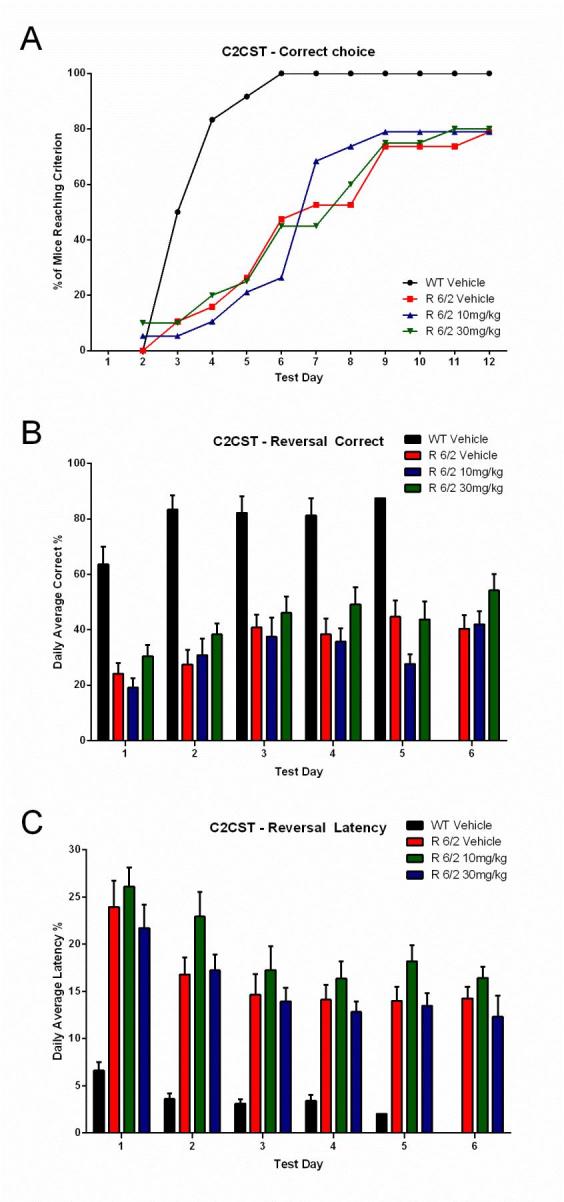
(A) Learning phase; data expressed as cumulative number of animals reaching criteria; (B) Reversal learning; data expressed as % correct choices made over time after platform reversal; (C) Latency to make the choice. Administration of SCH51866 did not improve any of these parameters. Deficits in R6/2 versus wildtype animals are clearly detectable at 9 weeks of age.

**MRI evaluation of regional brain volume loss and survival after chronic administration of SCH-51866 in R6/2 mice.**
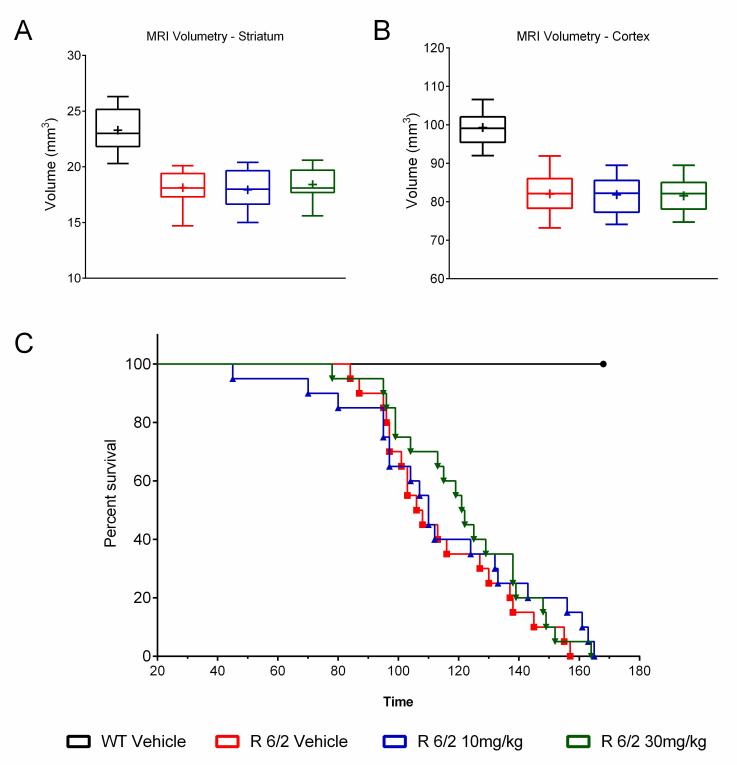
There was no improvement in brain volume in R6/2 treated striatum (A) or cortex (B); (C) Survival analysis of mice treated with SCH-51866 versus R6/2 vehicle-treated animals and WT controls showed no survival benefit of SCH-51866 treatment.

**Table 3 - Summary of statistical values obtained in various efficacy endpoints in R6/2 mice.** ▲ = significant enhancement compared to Vehicle-treated R6/2 mice (One-way ANOVA, Dunnett’s post-hoc); ▼ = significant impairment compared to Vehicle-treated R6/2 mice (One-way ANOVA, Dunnett’s post-hoc); p>0.05 = no significant differences compared to Vehicle-treated R6/2 mice (One-way ANOVA).

	10 mg/kg SCH-51866 (p.o., BID)	30 mg/kg SCH-51866 (p.o., BID)
**Body Weight**		
Age Week 4	ANOVA: p=0.878	ANOVA: p=0.878
Age Week 5	ANOVA: p=0.762	ANOVA: p=0.762
Age Week 6	ANOVA: p=0.934	ANOVA: p=0.934
Age Week 7	ANOVA: p=0.766	ANOVA: p=0.766
Age Week 8	ANOVA: p=0.926	ANOVA: p=0.926
Age Week 9	ANOVA: p=0.886	ANOVA: p=0.886
Age Week 10	ANOVA: p=0.969	ANOVA: p=0.969
Age Week 11	ANOVA: p=0.891	ANOVA: p=0.891
Age Week 12	ANOVA: p=0.774	ANOVA: p=0.774
Age Week 13	ANOVA: p=0.855	ANOVA: p=0.855
Age Week 14	ANOVA: p=0.878	ANOVA: p=0.878
Age Week 15	ANOVA: p=0.989	ANOVA: p=0.989
Age Week 16	ANOVA: p=0.651	ANOVA: p=0.651
Age Week 17	ANOVA: p=0.817	ANOVA: p=0.817
Age Week 18	ANOVA: p=0.644	ANOVA: p=0.644
Age Week 19	ANOVA: p=0.975	ANOVA: p=0.975
Age Week 20	ANOVA: p=0.900	ANOVA: p=0.900
Age Week 21	ANOVA: p=0.996	ANOVA: p=0.996
**Rotarod**		
Age Week 4	ANOVA: p=0.458	ANOVA: p=0.458
Age Week 6	ANOVA: p=0.262	ANOVA: p=0.262
Age Week 8	ANOVA: p=0.171	ANOVA: p=0.171
**Grip Strength **		
Age Week 4	ANOVA: p=0.612	ANOVA: p=0.612
Age Week 12	p=0.860	▲p=0.006
**Open Field **		
Total Distance		
- Age Week 4	ANOVA: p=0.792	ANOVA: p=0.792
- Age Week 6	ANOVA: p=0.101	ANOVA: p=0.101
- Age Week 8	ANOVA: p=0.333	ANOVA: p=0.333
- Age Week 12	p=0.454	▼p=0.016
Center Distance		
- Age Week 4	ANOVA: p=0.701	ANOVA: p=0.701
- Age Week 6	ANOVA: p=0.305	ANOVA: p=0.305
- Age Week 8	ANOVA: p=0.693	ANOVA: p=0.693
- Age Week 12	p=0.112	▼p=0.012
Rearing Rate in Center		
- Age Week 4	ANOVA: p=0.341	ANOVA: p=0.341
- Age Week 6	ANOVA: p=0.594	ANOVA: p=0.594
- Age Week 8	ANOVA: p=0.519	ANOVA: p=0.519
- Age Week 12	ANOVA: p=0.423	ANOVA: p=0.423
Total Rearing Frequency		
- Age Week 4	ANOVA: p=0.542	ANOVA: p=0.542
- Age Week 6	ANOVA: p=0.639	ANOVA: p=0.639
- Age Week 8	ANOVA: p=0.395	ANOVA: p=0.395
- Age Week 12	ANOVA: p=0.703	ANOVA: p=0.703
Average Velocity		
- Age Week 4	ANOVA: p=0.461	ANOVA: p=0.461
- Age Week 6	ANOVA: p=0.686	ANOVA: p=0.686
- Age Week 8	ANOVA: p=0.138	ANOVA: p=0.138
- Age Week 12	ANOVA: p=0.197	ANOVA: p=0.197
**Rear Climbing Test**		
Rearing Frequency		
- Age Week 4	p=0.507	p=0.186
- Age Week 6	ANOVA: p=0.203	ANOVA: p=0.203
- Age Week 8	ANOVA: p=0.782	ANOVA: p=0.782
- Age Week 12	ANOVA: p=0.122	ANOVA: p=0.122
Latency to Climb		
- Age Week 4	ANOVA: p=0.365	ANOVA: p=0.365
- Age Week 6	ANOVA: p=0.257	ANOVA: p=0.257
- Age Week 8	ANOVA: p=0.193	ANOVA: p=0.193
- Age Week 12	ANOVA: p=0.846	ANOVA: p=0.846
Time Spent Climbing		
- Age Week 4	ANOVA: p=0.853	ANOVA: p=0.853
- Age Week 6	ANOVA: p=0.291	ANOVA: p=0.291
- Age Week 8	ANOVA: p=0.750	ANOVA: p=0.750
- Age Week 12	ANOVA: p=0.724	ANOVA: p=0.724
Climbing Instances		
- Age Week 4	ANOVA: p=0.484	ANOVA: p=0.484
- Age Week 6	ANOVA: p=0.120	ANOVA: p=0.120
- Age Week 8	ANOVA: p=0.166	ANOVA: p=0.166
- Age Week 12	ANOVA: p=0.671	ANOVA: p=0.671
**Cued 2-CST at 9 Weeks**		
Acquisition: % of Mice Reaching Criterion		
	Fisher's exact test:	Fisher's exact test:
- Test Day 2	p>0.999	p=0.487
- Test Day 3	p>0.999	p>0.999
- Test Day 4	p>0.999	p>0.999
- Test Day 5	p>0.999	p>0.999
- Test Day 6	p=0.313	p>0.999
- Test Day 7	p=0.508	p=0.752
- Test Day 8	p=0.313	p=0.751
- Test Day 9	p>0.999	p>0.999
- Test Day 10	p>0.999	p>0.999
- Test Day 11	p>0.999	p=0.716
- Test Day 12	p>0.999	p>0.999
Reversal: Correct Choice		
- Test Day 1	ANOVA: p=0.117	ANOVA: p=0.117
- Test Day 2	ANOVA: p=0.311	ANOVA: p=0.311
- Test Day 3	ANOVA: p=0.573	ANOVA: p=0.573
- Test Day 4	ANOVA: p=0.206	ANOVA: p=0.206
- Test Day 5	ANOVA: p=0.057	ANOVA: p=0.057
- Test Day 6	ANOVA: p=0.140	ANOVA: p=0.140

**Table 4 - Summary of the survival study in R6/2 mice.** 

	Vehicle (p.o., BID) (30% Propylene Glycol, 20% Solutol HS15, 50% 50nM Citrate)	10 mg/kg (p.o., BID) SCH-51866	30 mg/kg (p.o., BID) SCH-51866
Mean (±SEM) / Medial Survival Time (days)	115 (±5) / 106	115 (±7) / 110	122 (±5) / 121
Log-rank (Peto) Test	Chi-square for equiv. of death rates p=0.555 Hazard ratio vs. other groups: vs. 10 mg/kg = 1.340 vs. 30 mg/kg = 1.325	Chi-square for equiv. of death rates p=0.555 Hazard ratio vs. other groups: vs. Vehicle = 1.340 vs. 30 mg/kg = 0.988	Chi-square for equiv. of death rates p=0.555 Hazard ratio vs. other groups: vs. Vehicle = 1.325 vs. 10 mg/kg = 0.988
Generalized Wilcoxon (Peto-Prentice) Test	Chi-square for equiv. of death rates p=0.543	Chi-square for equiv. of death rates p=0.543	Chi-square for equiv. of death rates p=0.543

For molecular analysis of gene expression changes and measurements of aggregate load (Figure 9) after chronic administration of SCH-51866, samples were collected from a satellite group of dosed animals at 13 weeks of age (n=6/treatment group in the lower dose group study). Overall, SCH-51866 had no effect on the aggregate load in the cortex and hippocampus when measured by the Seprion ligand ELISA (Figure 9A). Also, the drug did not have a statistically significant effect on the expression of disease-relevant genes, monitored in striatum, cortex and cerebellum (Figure 9B-D).

**Evaluation of HTT aggregate load, and markers of transcriptional dysregulation, after chronic administration of SCH-51866 in R6/2 animals.**
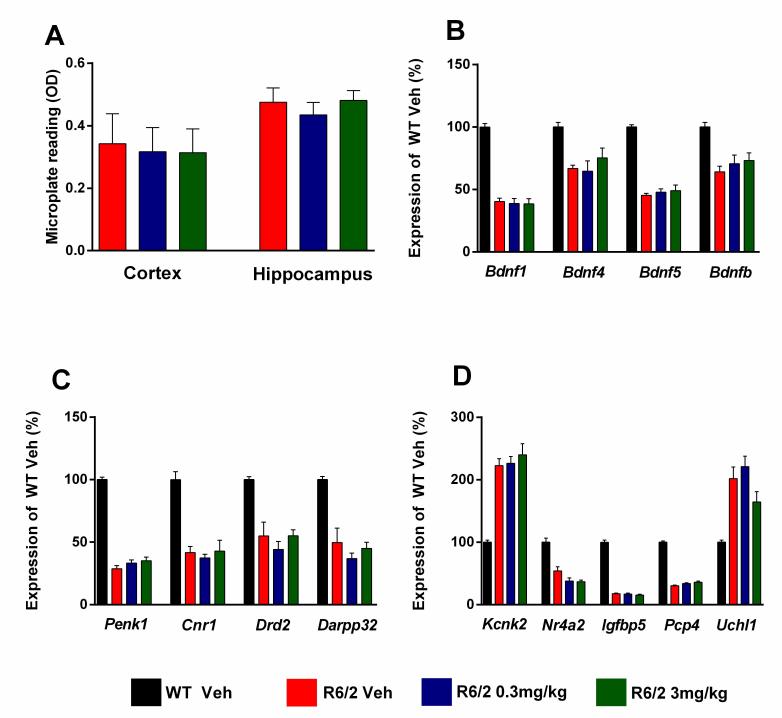
A) Analysis of cortical and hippocampal aggregated HTT levels as measured using the Seprion ligand in R6/2 animals dosed with vehicle or SCH-51866. (B-C) Analysis of specified gene expression changes in R6/2s vs control littermates and in R6/2s treated chronically with SCH-51866 in B) cortex, C) striatum, and D) cerebellum. The drug had no effects on the dysregulation of these genes. Bdnf = brain derived neurotrophic factor, promoter transcripts 1, 4 and 5 and coding region (b); Penk1 = preproenkephalin; Cnr1 = cannabinoid receptor 1; Drd2 = dopamine receptor 2; Darpp32 = dopamine and cAMP regulated neuronal phosphoprotein; Kcnk2 = potassium channel, subfamily K member 2; Nr4a2 = nuclear receptor subfamily 4 group A member 2; Igfbp5 = insulin-like growth factor binding protein; Pcp4 = Purkinje cell protein 4; Uchl1 = ubiquitin carboxy-terminal hydrolase L1

Overall, despite the confirmation of adequate target engagement we found very little evidence for a beneficial role for SCH-51866 in disease progression, including motor and cognitive behavior, brain volumetric changes, amelioration of disease-relevant gene deregulation, or HTT aggregate load in R6/2 mice. The only significant finding was an improvement in grip strength in the highest dose group of R6/2 mice at 12 weeks of age. However, this improvement was offset by a modest but significant worsening of performance in open field evaluation at this dose and age. While neither inhibiting PDE1 activity nor elevating cGMP levels in the striatum of R6/2 mice elicited detrimental effects, our results collectively suggest that PDE1 is not a target of further interest for the treatment of HD.

## Conflict of Interest

Psychogenics, Charles River Discovery Research Services, Brains On-Line, and Neuroservice conducted the research described through a fee-for-service agreement for CHDI Foundation. Work conducted at GB’s laboratory was funded by a contract with CHDI Foundation. As employees of CHDI Management and advisors to CHDI Foundation, VB, LP, MB, CD, and IM-S were all intimately involved in the study design, data collection and analysis, decision to publish, and preparation of the manuscript.

## Correspondence

Ignacio Munoz-Sanjuan, email: ignacio.munoz@chdifoundation.org

## Appendix 1

### Supplementary Report 1

Download PDF

### Supplementary Report 2

Download PDF
